# Reliability of Corneal Dynamic Scheimpflug Analyser Measurements in Virgin and Post-PRK Eyes

**DOI:** 10.1371/journal.pone.0109577

**Published:** 2014-10-10

**Authors:** Xiangjun Chen, Aleksandar Stojanovic, Yanjun Hua, Jon Roger Eidet, Di Hu, Jingting Wang, Tor Paaske Utheim

**Affiliations:** 1 SynsLaser Kirurgi, Oslo and Tromsø, Norway; 2 University of Oslo, Oslo, Norway; 3 Eye Department, University Hospital of North Norway, Tromsø, Norway; 4 Department of Ophthalmology, Taihe Hospital, Hubei Medical University, Hubei, China; 5 School of Ophthalmology and Optometry and Eye Hospital, Wenzhou Medical University, Wenzhou, Zhejiang, China; 6 Department of Medical Biochemistry, Oslo University Hospital, Oslo, Norway; 7 Institute of Oral Biology, Faculty of Dentistry, University of Oslo, Oslo, Norway; Bascom Palmer Eye Institute, University of Miami School of Medicine;, United States of America

## Abstract

**Purpose:**

To determine the measurement reliability of CorVis ST, a dynamic Scheimpflug analyser, in virgin and post-photorefractive keratectomy (PRK) eyes and compare the results between these two groups.

**Methods:**

Forty virgin eyes and 42 post-PRK eyes underwent CorVis ST measurements performed by two technicians. Repeatability was evaluated by comparing three consecutive measurements by technician A. Reproducibility was determined by comparing the first measurement by technician A with one performed by technician B. Intraobserver and interobserver intraclass correlation coefficients (ICCs) were calculated. Univariate analysis of covariance (ANCOVA) was used to compare measured parameters between virgin and post-PRK eyes.

**Results:**

The intraocular pressure (IOP), central corneal thickness (CCT) and 1_st_ applanation time demonstrated good intraobserver repeatability and interobserver reproducibility (ICC≧0.90) in virgin and post-PRK eyes. The deformation amplitude showed a good or close to good repeatability and reproducibility in both groups (ICC≧0.88). The CCT correlated positively with 1_st_ applanation time (r = 0.437 and 0.483, respectively, *p*<0.05) and negatively with deformation amplitude (r = −0.384 and −0.375, respectively, *p*<0.05) in both groups. Compared to post-PRK eyes, virgin eyes showed longer 1_st_ applanation time (7.29±0.21 vs. 6.96±0.17 ms, *p*<0.05) and lower deformation amplitude (1.06±0.07 vs. 1.17±0.08 mm, *p<0.05*).

**Conclusions:**

CorVis ST demonstrated reliable measurements for CCT, IOP, and 1_st_ applanation time, as well as relatively reliable measurement for deformation amplitude in both virgin and post-PRK eyes. There were differences in 1_st_ applanation time and deformation amplitude between virgin and post-PRK eyes, which may reflect corneal biomechanical changes occurring after the surgery in the latter.

## Introduction

The cornea is a viscoelastic structure with quantifiable biomechanical properties [1]. These properties are related to corneal thickness, age, intraocular pressure (IOP), hydration, and various pathologies [2]–[5]. The cornea's biomechanical behaviour is mostly dictated by the stroma, which encompasses 90% of the total corneal thickness and has a greater mechanical stiffness than the other corneal layers [6].

Corneal biomechanical failure is the basis of keratectatic diseases [7] such as keratoconus and pellucid marginal degeneration. The ability to quantify corneal biomechanical failure represents an important step towards better understanding and treatment of keratectatic diseases. In addition, corneal refractive laser ablation in virgin eyes weakens the cornea mechanically due to tissue removal, leading to deterioration in corneal biomechanical strength [8]. Biomechanical changes may also affect the refractive outcome [9]. Moreover, biomechanical weakening after corneal refractive laser treatment may potentially induce iatrogenic keratectasia [10]. Therefore, knowledge of corneal biomechanical properties is important in predicting clinical outcomes [11] and in identifying cases with high risk for postoperative keratectasia after corneal refractive surgery.

Most of the earlier studies concerning corneal biomechanical properties were performed *in vitro* [12]–[14]. The Ocular Response Analyser (ORA, Reichert, Inc., Depew, NY) was the first device available to evaluate *in vivo* corneal biomechanical response to an air-puff [1]. It employs a quantitative electro-optical system to monitor the pressures at which the cornea flattens inward and outward by registering the corneal reflex of infrared light. The recently introduced ultra-high-speed Scheimpflug video-imaging device (CorVis ST; Oculus, Wetzlar, Germany) is the first instrument allowing visualization and measurement of corneal deformation in response to a standardized air-puff pressure. Data evaluating the intraobserver repeatability and interobserver reproducibility of measurements with this relatively new device are scarce [15], [16]. Furthermore, such studies as are available concern only healthy virgin eyes. The main goal of the present study was to test the hypothesis that the CorVis ST performs reliable measurements in both virgin and post-refractive surgery eyes. To our knowledge, this is the first study to evaluate the repeatability and reproducibility of CorVis ST measurements in post-refractive surgery eyes. The secondary purpose was to test the hypothesis that the measurements can reveal differences in biomechanical properties between these two groups.

### CorVis ST

The CorVis ST utilizes an ultraviolet free blue (455 nm wavelength) light emitting diode (LED) and an ultra-high-speed (4330 frames per second) Scheimpflug camera to record the corneal deformation response to a high intensity air impulse. The air impulse originates from a metered, symmetrical, and fixed maximal internal pump generating a pressure of 25 kilopascal [16]. When the eye is aligned and the Scheimpflug image is in focus, the air puff gets released automatically and the cornea is imaged during the deformation event. The air pulse (lasting approximately 20 ms) forces the cornea inwards through applanation until it achieves its highest concavity (concavity phase). On its way back, the cornea undergoes a second applanation before achieving its natural shape.

A total of approximately 140 images of the cornea's two-dimensional cross-section are collected. By software tracing of the anterior and posterior corneal boundaries in individual image frames, parameters describing the corneal deformation response are automatically generated by the instrument. The CorVis ST software version 1.00r30 rev. 771 was used in the current study.

**Figure 1:**
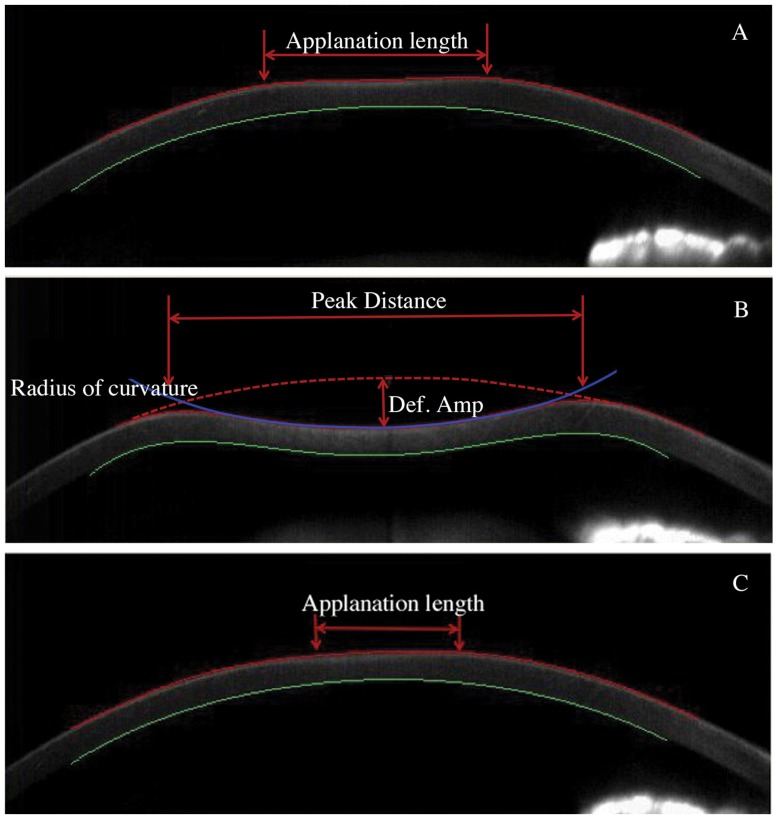
The CorVis ST utilizes the Scheimpflug camera to record the dynamic procedure of the corneal response to an air puff. A) The 1_st_ applanation is achieved. B) The cornea reaches its highest concavity. C) The 2_nd_ applanation is achieved when the cornea rebounds to its original position from the highest concavity.

With the Corvis ST the biomechanical response of the cornea is characterized by three phases: 1_st_ applanation, highest concavity, and 2_nd_ applanation. In addition to intraocular pressure (IOP) and central corneal thickness (CCT) values, time (time to reach applanation), length (the length of the flattened central cornea), and velocity (the velocity of the corneal apex movement during applanation) at the moment of both the 1_st_ and 2_nd_ applanation events are recorded. The following characteristics at the point of highest concavity are also presented: the highest concavity time, the deformation amplitude, the distance between bending points of the cornea (peak distance), and the concave radius of curvature. (Figure 1.)

## Patients and Methods

Forty candidates for laser refractive surgery (virgin-eye group: 28 males and 12 females) and 42 subjects treated for myopia and astigmatism with photorefractive keratectomy (PRK) earlier (post-PRK group: 23 males and 19 females) were recruited. The PRK treatments were performed using topography-guided transepithelial surface ablation with the iRES system (iRES, iVIS Technology, Taranto, Italy) at SynsLaser Clinic in Tromsø, Norway, 12.69±10.08 months (range: 2 to 48) prior to the current examination. All participants received an extensive ophthalmic examination including Placido-based topography (Nidek OPD Scan II, Nidek Co. Ltd., Aichi, Japan), Scheimpflug topo/tomography (Precisio, iVIS Technology, Taranto, Italy), slit-lamp biomicroscopy and tonometry (Icare tonometer, Revenio Group Corporation, Helsinki, Finland) to exclude corneal and other ocular pathologies. The Regional Committee for Medical and Health Research Ethics in Norway approved the study entitled "2013/762 - Biomechanical cornea measurements by means of CorVis ST". The research complied with the tenets of the Declaration of Helsinki and written informed consent was obtained from each participant before examination. Only the data from the right eye of each participant was used for the present study.

The CorVis ST measurements were performed three times by technician A and one time by technician B. The measurement sequence between the technicians was randomized using a randomization table. A one-minute pause was given between each measurement. Repeatability was evaluated by comparing the three consecutive measurements performed by technician A. Reproducibility was determined by comparing the first measurement by technician A with the one performed by technician B. Mean CorVis ST measured values obtained from the three measurements by technician A were used to compare the differences between the virgin and post-PRK eyes groups, as well as for the correlation analysis.

### Statistical Analysis

MedCalc software 11.4.2 (MedCalc Software, Ostend, Belgium) and SPSS for Mac software (version 19. SPSS, Inc) were used for statistical analysis. A *p*-value of less than 0.05 was considered statistically significant. Descriptive statistical results were expressed as mean ± standard deviation (SD). The within-subject standard deviation (S_w_), within-subject coefficient of variation (COV), and intraclass correlation coefficient (ICC) were determined to assess the intraobserver repeatability. Interobserver S_w_, COV, and ICC were calculated to assess interobserver reproducibility. Independent sample *t*-test was used to compare the CorVis measured parameters in virgin and post-PRK eyes groups. For the parameters that showed significant differences, univariate analysis of covariance (ANCOVA) was then applied to adjust for selected covariates (age, CCT measured by the CorVis ST, and mean simulated keratometry (simK) value measured by OPD Scan II) to control for potentially confounding factors. Pearson or Spearman correlations were applied to examine the relationship between CCT, manifest refraction spherical equivalent (MRSE) and the deformation parameters.

## Results

### Patient Demographics

The mean age of the participants at the time of the examination was 27.6±9.0 (range, 18 to 48) and 31.8±6.7 years (range, 20 to 48) for the virgin-eye and post-PRK groups, respectively. In the virgin-eye group, the values of central corneal thickness (CCT) measured by Precisio, IOP measured by Icare rebound tonometer, and mean simK measured by OPD Scan II were not significantly different from the preoperative values of the post-PRK group (Table 1). The mean manifest refraction spherical equivalent (MRSE) in the virgin-eye and its preoperative value in the post-PRK groups were −2.15±2.28 D and −3.52±1.93 D, respectively. In the post-PRK group, the mean maximum ablation depth was 66.71±27.84* µm* (range: 18 to 129).

**Table 1:** Demographic Data of Participants.

	Virgin eyes (n = 40)	Post-PRK eyes (n = 42)	*p*
**Age, years**	27.9±9.0 (18, 48)	31.8±6.9 (20, 48)	0.03
**CCT (Precisio), ** ***µm***	547.82±26.78	preop 542.02±30.68 postop 485.00±40.10	0.30* 0.000*
**IOP (Icare), mmHg**	15.20±2.57	preop 15.81±3.29 postop 12.71±2.77	0.46* 0.000*
**MRSE, D**	−2.15±2.28	preop −3.52±1.93 postop 0.01±0.48	0.03* 0.000*
**Mean simK (OPD Scan II), D**	43.47±1.38	preop 43.81±1.58 postop 40.87±1.63	0.18* 0.000*

CCT =  central corneal thickness; IOP =  intraocular pressure; MRSE =  manifest refraction spherical equivalent; simK =  simulated karatometry.

* *p* values were adjusted for age-difference.

### Intraobserver Repeatability and Interobserver Reproducibility

Tables 2 and 3 present the intraobserver repeatability of the CorVis ST measurements. In the virgin-eye group, the IOP, CCT, 1_st_ applanation time, and 2_nd_ applanation time demonstrated good repeatability (ICC≧0.92), followed by deformation amplitude (ICC: 0.88), Radius of Curvature (ICC: 0.70), 2_nd_ applanation velocity (ICC: 0.65), and highest concavity time (ICC: 0.64). The other parameters showed poor repeatability with large COVs and low ICCs. In the post-PRK group, the IOP, CCT, 1_st_ applanation time, and deformation amplitude demonstrated good repeatability (ICC≧0.90), followed by 2_nd_ applanation time (ICC: 0.89), 2_nd_ applanation velocity (ICC: 0.79), highest concavity time (ICC: 0.66), and radius of curvature (ICC: 0.63). The other parameters showed poor repeatability with large COVs and low ICCs.

**Table 2:** Intraobserver Repeatability of Parameters Obtained by Corvis in Virgin-Eye Group (n = 40).

Parameters	Mean ± SD	Sw	2.77Sw	COV (%)	ICC (95%CI)
IOP (mmHg)	14.46±1.33	0.59	1.62	3.59	0.93 (0.89∼0.96)
CCT (*µm*)	543.32±25.08	5.34	12.56	0.69	0.99 (0.98∼0.99)
1_st_ appl. time (ms)	7.29±0.21	0.09	0.24	1.09	0.94 (0.90∼0.97)
1_st_ appl. length (mm)	1.83±0.18	0.29	0.81	13.94	0.10 (−0.52∼0.49)
1_st_ appl. velocity (m/s)	0.14±0.02	0.03	0.08	18.82	0.25 (−0.26∼0.57)
2_nd_ appl. time (ms)	21.65±0.34	0.17	0.48	0.71	0.92 (0.87∼0.95)
2_nd_ appl. length (mm)	1.89±0.29	0.45	1.24	21.53	0.17 (−0.39∼0.53)
2_nd_ appl. velocity (m/s)	−0.34±0.04	0.04	0.10	−9.77	0.65 (0.42∼0.80)
Highest concavity time (ms)	16.40±0.37	0.37	1.02	2.02	0.64 (0.40∼0.80)
Peak distance (mm)	4.36±0.66	1.17	3.23	21.80	−0.04 (−0.74∼0.41)
Radius of curvature (mm)	7.49±0.60	0.55	1.51	6.31	0.70 (0.49∼0.83)
Deformation amplitude (mm)	1.06±0.07	0.04	0.11	3.34	0.88 (0.81∼0.93)

SD  =  standard deviation, ICC  =  intraclass correlation coefficient, CI  =  confidence interval, Sw  =  within-subject standard deviation, COV  =  within-subject coefficient of variation, IOP  =  intraocular pressure, CCT  =  central corneal thickness.

**Table 3:** Intraobserver Repeatability of Parameters Obtained by Corvis in Post-PRK Group (n = 42).

Parameters	Mean ± SD	Sw	2.77Sw	COV (%)	ICC (95%CI)
IOP (mmHg)	12.38±1.02	0.55	1.52	3.85	0.90 (0.84∼0.94)
CCT (µm)	481.18±42.45	8.16	22.61	2.29	0.99 (0.98∼0.99)
1_st_ appl. time (ms)	6.96±0.17	0.09	0.25	1.11	0.91 (0.85∼0.95)
1_st_ appl. length (mm)	1.82±0.22	0.36	0.99	17.65	0.08 (−0.54∼0.47)
1_st_ appl. velocity (m/s)	0.13±0.02	0.03	0.09	23.30	−0.27 (−1.14∼0.28)
2_nd_ appl. time (ms)	21.96±0.31	0.18	0.49	0.69	0.89 (0.81∼0.94)
2_nd_ appl. length (mm)	1.70±0.39	0.49	1.37	26.01	0.48 (0.12∼0.70)
2_nd_ appl. velocity (m/s)	−0.40±0.06	0.04	0.12	−10.35	0.79 (0.65∼0.88)
Highest concavity time (ms)	16.48±0.35	0.41	1.15	2.12	0.66 (0.43∼0.81)
Peak distance (mm)	4.56±0.77	1.12	3.10	18.34	0.30 (−0.18∼0.60)
Radius of curvature (mm)	6.43±0.66	0.70	1.94	6.76	0.63 (0.38∼0.79)
Deformation amplitude (mm)	1.17±0.08	0.04	0.12	3.16	0.92 (0.86∼0.95)

SD  =  standard deviation, ICC  =  intraclass correlation coefficient, CI  =  confidence interval, Sw  =  within-subject standard deviation, COV  =  within-subject coefficient of variation, IOP  =  intraocular pressure, CCT  =  central corneal thickness.

When comparing the interobserver reproducibility of the CorVis ST parameters, the IOP, CCT, 1_st_ applanation time, and 2_nd_ applanation time demonstrated good reproducibility (ICC≧0.91), followed by deformation amplitude (ICC: 0.88), radius of curvature (ICC: 0.64) and 2_nd_ applanation velocity (ICC: 0.59) in the virgin-eye group. In the post-PRK group, the IOP, CCT, and 1_st_ applanation time demonstrated good reproducibility (ICC≥0.90), followed by deformation amplitude (ICC: 0.88), radius of curvature (ICC: 0.83), 2_nd_ applanation time (ICC: 0.79), highest concavity time (ICC: 0.63), 2_nd_ applanation velocity (ICC: 0.60), and 2_nd_ applanation length (ICC: 0.52), (Table 4 and 5). The other parameters showed poor reproducibility.

**Table 4:** Interobserver Reproducibility of Parameters Obtained by Corvis in Virgin-Eye Group (n = 40).

Parameters	Mean Difference ± SD	Sw	2.77Sw	COV (%)	ICC (95%CI)
IOP (mmHg)	−0.01±0.82	0.58	1.60	3.25	0.92 (0.86∼0.96)
CCT (µm)	−1.49±6.76	4.78	13.24	0.72	0.98 (0.97∼0.99)
1_st_ appl. time (ms)	0.01±0.12	0.08	0.23	0.96	0.93 (0.88∼0.96)
1_st_ appl. length (mm)	−0.02±0.38	0.27	0.73	11.67	0.29 (−0.35(0.63)
1st appl. velocity (m/s)	(0.005±0.05	0.03	0.09	19.10	0.08 ((0.75(0.51)
2nd appl. time (ms)	(0.01±0.24	0.17	0.47	0.61	0.91 (0.82(0.95)
2nd appl. length (mm)	(0.10±0.58	0.44	1.14	17.37	0.12 ((0.65(0.53)
2nd appl. velocity (m/s)	(0.01±0.06	0.04	0.11	(9.63	0.59 (0.25(0.78)
Highest concavity time (ms)	(0.02±0.54	0.38	1.06	1.81	0.47 (0.00(0.72)
Peak distance (mm)	0.11±1.47	1.04	2.89	13.08	0.06 ((1.45(0.63)
Radius of curvature (mm)	0.01±0.81	0.57	1.58	5.01	0.64 (0.31∼0.81)
Deformation amplitude (mm)	−0.002±0.06	0.04	0.11	1.95	0.88 (0.78∼0.94)

SD  =  standard deviation, ICC  =  intraclass correlation coefficient, CI  =  confidence interval, Sw  =  within-subject standard deviation, COV  =  within-subject coefficient of variation, IOP  =  intraocular pressure, CCT  =  central corneal thickness.

**Table 5:** Interobserver Reproducibility of Parameters Obtained by Corvis in Post-PRK Group (n = 42).

Parameters	Mean Difference ± SD	Sw	2.77Sw	COV (%)	ICC (95%CI)
IOP (mmHg)	−0.17±0.70	0.50	1.38	2.81	0.90 (0.81∼0.95)
CCT (µm)	0.43±5.05	3.57	9.89	0.58	1.00 (0.99∼1.00)
1_st_ appl. time (ms)	−0.02±0.11	0.08	0.23	0.84	0.90 (0.82∼0.95)
1_st_ appl. length (mm)	0.08±0.46	0.32	0.90	14.45	0.27 (−0.36∼0.60)
1_st_ appl. velocity (m/s)	−0.01±0.04	0.03	0.09	17.54	0.45 (−0.03∼0.70)
2_nd_ appl. time (ms)	0.04±0.28	0.20	0.55	0.64	0.79 (0.61∼0.89)
2_nd_ appl. length (mm)	−0.11±0.64	0.45	1.25	20.23	0.52 (0.12∼0.74)
2_nd_ appl. velocity (m/s)	0.01±0.07	0.05	0.15	−11.03	0.60 (0.26∼0.78)
Highest concavity time (ms)	0.05±0.49	0.35	0.97	1.54	0.63 (0.31∼0.80)
Peak distance (mm)	0.001±1.57	1.11	3.08	15.23	0.26 (−0.39∼0.61)
Radius of curvature (mm)	−0.06±0.49	0.35	0.97	4.27	0.83 (0.68∼0.91)
Deformation amplitude (mm)	−0.02±0.15	0.04	0.12	3.89	0.88 (0.78∼0.94)

SD  =  standard deviation, ICC  =  intraclass correlation coefficient, CI  =  confidence interval, Sw  =  within-subject standard deviation, COV  =  within-subject coefficient of variation, IOP  =  intraocular pressure, CCT  =  central corneal thickness.

The IOP, CCT, and 1_st_ applanation time demonstrated good intraobserver repeatability and interobserver reproducibility in both groups. The 2_nd_ applanation time had good repeatability and reproducibility in the virgin eyes, with close to good repeatability but not good reproducibility in post-PRK eyes. The deformation amplitude showed a good or close to good repeatability and reproducibility in both groups.

### Comparison of the Measurements between Virgin-Eye and Post-PRK Groups

Differences in the CorVis ST measured parameters between the virgin and post-PRK eyes are listed in Table 6. After adjustment for age, CCT, and mean simK, the differences in the mean values of IOP, 1_st_ applanation time, 2_nd_ applanation time, radius of curvature, and deformation amplitude remained significant. Compared to the virgin-eye group, the post-PRK group demonstrated a shorter 1_st_ applanation time, longer 2_nd_ applanation time, smaller radius of curvature, and larger deformation amplitude. The CCT demonstrated a confounding effect in the above-mentioned parameters (*p*<0.05 in all analyses), while age and simK did not show statistically significant confounding effects (*p*>0.05 in all analyses).

**Table 6:** Comparison of The CorVis ST Measurments Between Virgin-Eye and Post-PRK Groups.

	Virgin-eye (n = 40)	post-PRK (n = 42)	Difference (mean ± SE)	*p*	Adjusted p*	Adjusted R^2^
**IOP (mmHg)**	14.46±1.33	12.38±1.02	2.08±0.26	0.000	0.002	0.520
**CCT (µm)**	543.32 ±25.08	485.41±39.00	57.90±7.28	0.000		
**1_st_ applanation**						
**Time (ms)**	7.29±0.21	6.96±0.17	0.33±0.04	0.000	0.003	0.519
**Length (mm)**	1.83±0.18	1.81±0.22	0.01±0.04	0.832		
**Velocity (m/s)**	0.14±0.02	0.13±0.02	0.01±0.00	0.010	0.614	0.160
**2_nd_ applanation**						
**Time (ms)**	21.65±0.34	21.96±0.31	−0.31±0.07	0.000	0.032	0.221
**Length (mm)**	1.89±0.29	1.70±0.39	0.19±0.08	0.013	0.958	0.133
**Velocity (m/s)**	−0.34±0.04	−0.40±0.06	0.07±0.01	0.000	0.053	0.372
**Highest concavity**						
**Time (ms)**	16.40±0.37	16.48±0.41	−0.07±0.09	0.374		
**Peak distance**	4.36±0.66	4.56±0.77	−0.20±0.16	0.216		
**Radius (mm)**	7.49±0.60	6.43±0.66	1.06±0.14	0.000	0.001	0.551
**Deformation amplitude (mm)**	1.06±0.07	1.17±0.08	−0.10±0.02	0.000	0.005	0.402

SE =  standard error, IOP  =  intraocular pressure, CCT  =  central corneal thickness.

p* values were adjusted for the effect of the age, CCT, mean simK difference between the virgin-eye and post-PRK groups.

Central corneal thickness measured with the CorVis ST correlated to IOP, 1_st_ applanation time, radius of curvature, and deformation amplitude (r = 0.439, 0.437, 0.357, and −0.384, respectively, *p*<0.05), without significant correlation to other parameters in the virgin-eye group. In the post-PRK group, it correlated to IOP, 1_st_ applanation time, 1_st_ applanation velocity, 2_nd_ applanation length, 2_nd_ applanation velocity, radius of curvature, and deformation amplitude (r = 0.482, 0.483, 0.401, 0.440, 0.395, 0.583, −0.375, respectively, *p*<0.05). The MRSE correlated to IOP, 1_st_ applanation time, and 2_nd_ applanation time, without significant correlation to other parameters in the virgin-eye group (r = −0.485, −0.492, and 0.420, respectively, *p*<0.05). The postoperative MRSE was found to correlate only to radius of curvature in the post-PRK group (r = 0.583, *p*<0.05).

## Discussion

*In vitro* experiments [12], [13] as well as theoretical mathematical models [17], [18] have demonstrated that the cornea exhibits both elastic and viscoelastic properties. When loaded, the cornea shows instantaneous deformation (purely elastic behaviour) followed by a time-dependent deformation response (viscoelastic behaviour) [19]. The ideal device for measuring corneal biomechanical properties *in vivo* should be accurate, provide repeatable and reproducible results, and be minimally invasive. In the current study, the intraobserver repeatability and interobserver reproducibility of CorVis ST measurements in virgin eyes and post-PRK eyes were investigated.

Similar to the studies performed by Nemeth *et al.* [16] and Hon *et al*. [15], we found that the following parameters had the best repeatability in both groups: CCT, IOP, 1_st_ applanation time, and deformation amplitude. The current study also presented good repeatability for 2_nd_ applanation time. In addition, the ICCs in the current study were generally higher than in the mentioned studies for most of the parameters measured. The differences between the studies may be attributed to different patient populations and software versions. For example, in the study by Nemeth *et al*., the mean age was 61.24±15.72 years (95% CI: 57.62 to 64.86 years), while the population in the current study was much younger. In the study by Hon *et al*., the software did not offer values for radius of curvature and peak distance. When comparing reproducibility, Hon *et al*. found a statistically significant difference in the CCT measurement between the two sessions. However, the intersession difference was calculated by comparing the examinations performed in the morning (9:00–10:99 am) and afternoon (3:99–5:99 pm) by the same observer. This time difference may have affected the reproducibility evaluation, as corneal thickness demonstrates diurnal variation [20]. The other parameters measured with the CorVis ST did not show satisfactory reliability. The ICCs varied between the virgin and post-PRK eyes.

It is conceivable that the cornea would be more difficult to deform and would deform less in eyes with a greater CCT. In line with other studies [15], [21], we revealed a negative correlation between CCT and deformation amplitude in both groups. In addition, the CCT correlated positively with 1_st_ applanation time and radius of curvature in both virgin and post-PRK eyes. However, correlations between CCT and 1_st_ applanation velocity, 2_nd_ applanation time, length, and velocity were only found in post-PRK eyes. This may imply that CCT in normal virgin eyes does not introduce much variation to some of the CorVis ST parameters, while affecting those measurements in biomechanically compromised corneas. The MRSE in our virgin-eyes group demonstrated correlation with some of the parameters measured by CorVis ST. This may need to be taken into consideration if a database of “healthy corneas” is built for the purpose of identifying biomechanically weaker corneas.

The IOP measured with the CorVis ST was significantly lower in the post-PRK eye group compared to the virgin-eye group, while the historical preoperative data (IOP measured by Icare, CCT, and corneal curvature) of the post-PRK group showed no significant difference compared to the respective data in the virgin-eye group. The CorVis ST measurements in our post-PRK group were performed a minimum of two months postoperatively, by which time the patients had discontinued the use of local steroids for at least three weeks, to exclude a possible pharmacological effect on their IOP. Some studies have demonstrated that IOP measured with the CorVis ST is more reliable compared to Goldmann applanation tonometry (GAT) and Topcon noncontact tonometry in virgin eyes (Topcon CT-80A Computerized Tonometer; Topcon, Tokyo, Japan) [22]. Still, in the version of CorVis ST used in this study, IOP is calculated based on the timing of the 1_st_ applanation event and is not adjusted for corneal biomechanical properties. Both CCT and corneal biomechanical properties can affect IOP measurements, with the latter suggested to be more influential [18]. The difference in the CorVis ST measured IOP between the groups was most likely caused by changes in corneal biomechanical properties and CCT after PRK.

Interestingly, before being adjusted for age, CCT, and simK, the CorVis ST parameters that demonstrated differences between the virgin and post-PRK eyes (1_st_ applanation time, 1_st_ applanation velocity, 2_nd_ applanation time, 2_nd_ applanation velocity, deformation amplitude and radius of curvature) were the same parameters as those showing differences between normal eyes and keratoconus eyes in the study conducted by Ali *et al*. [23]. It seems that these parameters may be of value in evaluating corneal biomechanical properties.

The earlier start of the apex indentation (shorter 1_st_ applanation time) and greater deformation amplitude in post-PRK eyes indicates a lower resistance to deformation due to a decrease in corneal stiffness [24], [25]. Shen *et al*. [26] compared corneal deformation parameters after femtosecond laser small incision lenticule extraction (SMILE), laser-assisted sub-epithelial keratomileusis (LASEK), and femtosecond laser-assisted LASIK (FS-LASIK). They found greater deformation amplitude and shorter 1_st_ applanation time in the FS-LASIK group compared to the LASEK group. However, those parameters did not differ significantly between the SMILE and LASEK groups, or between SMILE and FS-LASIK groups. This indicates that corneal refractive surgery alters the stiffness of the cornea to different degrees with respect to different surgical approaches.

In the current study the CorVis ST measurements in virgin- and post-PRK eyes were taken from two groups of unrelated populations. Pre- and postoperative comparison of the same population would have been better suited to evaluate the changes in biomechanical properties caused by the surgery. We attempted to compensate for this by applying age, CCT, and simK as covariates to adjust for potential confounding factors. For the sake of this discussion we also introduced a separate group of 28 eyes of 16 patients who underwent PRK for myopic astigmatism (mean preoperative MRSE: −3.35±1.98 D, mean postoperative time 9.21±5.09 months) with both pre- and postoperative CorVis ST measurements. The pre- and postoperative CorVis ST measurements of CCT and IOP in that group [547.53±28.89 µm vs. 460.32±48.57 µm (*p*<0.05), and 15.00±1.48 mmHg vs. 13.48±1.24 mmHg (*p*<0.001), respectively] were similar to the differences found in the virgin and post-PRK eyes in the current study. Comparable similarity was also found for the 1_st_ applanation time [7.37±0.23 vs. 7.14±0.20 ms (*p*<0.001)], 2_nd_ applanation time (21.39±0.32 vs. 21.57±0.25 ms (*p*<0.05), radius of curvature [7.76±0.83 vs. 6.55±0.66 mm (*p<0.001*)] and deformation amplitude [1.03±0.08 vs. 1.10±0.08 mm, (*p*<0.05)]. Still, a separate study measuring pre- and post-PRK parameters with a larger population is warranted.

The current study demonstrated that, in addition to measurements of CCT and IOP, the CorVis ST showed relatively good reliability in measurements of 1_st_ applanation time and deformation amplitude in both virgin- and post-PRK eyes. The differences in 1_st_ applanation time and deformation amplitude between virgin and post-PRK eyes may imply that the CorVis ST's direct view of the corneal deformation may offer information that promises to yield clinically relevant parameters correlated with corneal biomechanical properties.
